# Impaired amino acid uptake leads to global metabolic imbalance of *Candida albicans* biofilms

**DOI:** 10.1038/s41522-022-00341-9

**Published:** 2022-10-13

**Authors:** Bettina Böttcher, Dominik Driesch, Thomas Krüger, Enrico Garbe, Franziska Gerwien, Olaf Kniemeyer, Axel A. Brakhage, Slavena Vylkova

**Affiliations:** 1 Septomics Research Center, Friedrich Schiller University and Leibniz Institute for Natural Product Research and Infection Biology—Hans Knöll Institute, Jena, Germany; 2 BioControl Jena, Jena, Germany; 3 Molecular and Applied Microbiology, Leibniz Institute for Natural Product Research and Infection Biology—Hans Knöll Institute, Jena, Germany; 4 Department of Microbiology and Molecular Biology, Institute of Microbiology, Friedrich Schiller University, Jena, Germany

**Keywords:** Biofilms, Pathogens

## Abstract

*Candida albicans* biofilm maturation is accompanied by enhanced expression of amino acid acquisition genes. Three state-of-the-art omics techniques were applied to detail the importance of active amino acid uptake during biofilm development. Comparative analyses of normoxic wild-type biofilms were performed under three metabolically challenging conditions: aging, hypoxia, and disabled amino acid uptake using a strain lacking the regulator of amino acid permeases Stp2. Aging-induced amino acid acquisition and stress responses to withstand the increasingly restricted environment. Hypoxia paralyzed overall energy metabolism with delayed amino acid consumption, but following prolonged adaptation, the metabolic fingerprints aligned with aged normoxic biofilms. The extracellular metabolome of *stp2*Δ biofilms revealed deficient uptake for 11 amino acids, resulting in extensive transcriptional and metabolic changes including induction of amino acid biosynthesis and carbohydrate and micronutrient uptake. Altogether, this study underscores the critical importance of a balanced amino acid homeostasis for *C. albicans* biofilm development.

## Introduction

The incidence of device-associated nosocomial infections is on the rise due to the increasing use of medical implants, especially in patients with compromised immune defenses. Such infections are mostly associated with biofilm formation, and *Candida albicans* is a prime example^1^. This pathogenic fungus consistently ranks among the top leading causes of catheter-associated infections^2,3^, which are difficult to treat due to the biofilm-associated high resistance to conventional antifungal therapy^4^.

A circuit of nine core and 50 auxiliary transcriptional factors control the structural integrity (adherence, filamentation), drug and stress resistance, and metabolism during *C. albicans* biofilm formation^5,6^. Among the metabolic regulators are factors with primary roles in glucose and amino acid metabolism, such as Gal4, Tye7 (control of glycolysis^7^), and Arg81, Gcn4, Stp2, and Leu3 (control of amino acid utilization and biosynthesis^6,8,9^), pointing towards the importance of metabolism in biofilm growth. Indeed, *C. albicans* biofilms exhibit characteristic transcriptional and metabolic features compared to planktonic cultures. Thus, biofilm maturation is accompanied by increased gene expression and higher metabolite abundance regarding glycolysis and reduction of TCA cycle and respiration metabolites^10,11^. These metabolic profiles resemble an oxygen deprivation response, indicative of the development of hypoxic niches within the biofilm^10–12^. Furthermore, robust biofilm growth is associated with an increased ability to metabolize certain amino acids instead of chained carbohydrates^13^. Indeed, several amino acid permease genes like *CAN2* are among the most induced transcripts during biofilm growth^5,14–16^. In a recent study, we described the important role of the transcriptional regulator of amino acid permeases, Stp2, in adherence and biofilm maturation^17^. Fully formed wild-type biofilms had an increased proportion of damaged or lysed cells compared to the *stp2*Δ biofilms with equal biomass, suggesting that reduced amino acid metabolism promotes biofilm longevity due to calorie restriction. These data underline that active amino acid uptake is required for *C. albicans* biofilm formation, but how this process contributes to biofilm maturation and how biofilm cells behave under restricted amino acid import remains unknown. We hypothesize that nutrient sensing and especially amino acid homeostasis play important roles to ensure adequate energy supply for biofilm growth^15,18–20^.

Our present work addresses the key questions of how the acquisition and metabolism of amino acids contribute to biofilm formation and how this nutritional homeostasis is balanced. We applied an integrative multi-omics approach to investigate metabolic processes accompanying biofilm maturation under oxygen or amino acid limitation (*stp2*Δ) to obtain a comprehensive picture of stress-adaptive strategies in biofilm growth. Our omics analysis revealed that biofilm cells are exposed to increasingly stressful conditions, making the acquisition of alternative nutrients and adaptation to oxygen limitation more essential. Mature wild-type biofilms are used as energy source amino acids released from lysed cells or extracellular polypeptides by secretion of proteases and up-regulated permease genes to facilitate sufficient uptake. Hypoxia-grown biofilms required a prolonged metabolic adaptation compared to their normoxic counterparts resulting in altered nutrient uptake, activation of glycolysis and TCA cycle gene expression, and overall impairment in energy production. Similar transcriptional responses were observed in aged normoxic biofilms. The Stp2-mediated uptake was assigned to a defined set of amino acids. Besides impaired amino acid utilization, the *stp2*Δ mutant biofilms showed changes in carbohydrate uptake, nitrogen metabolism, and macro- and micronutrient acquisition, demonstrating wide-ranging metabolic restructuring to maintain amino acid homeostasis, which is critical for *C. albicans* biofilm maintenance.

## Results

### *C. albicans* biofilms undergo starvation during maturation

To investigate the underlying metabolic and regulative processes of biofilm maturation and aging, *C. albicans* biofilms were sampled at 8, 24, and 48 h and analyzed for the transcriptome, the intracellular and extracellular metabolome, and the secreted proteome profile. Biofilms were cultivated in RPMI, a widely used host-like medium that is especially suitable for metabolomic analyses due to its defined formulation.

**Fig. 1:**
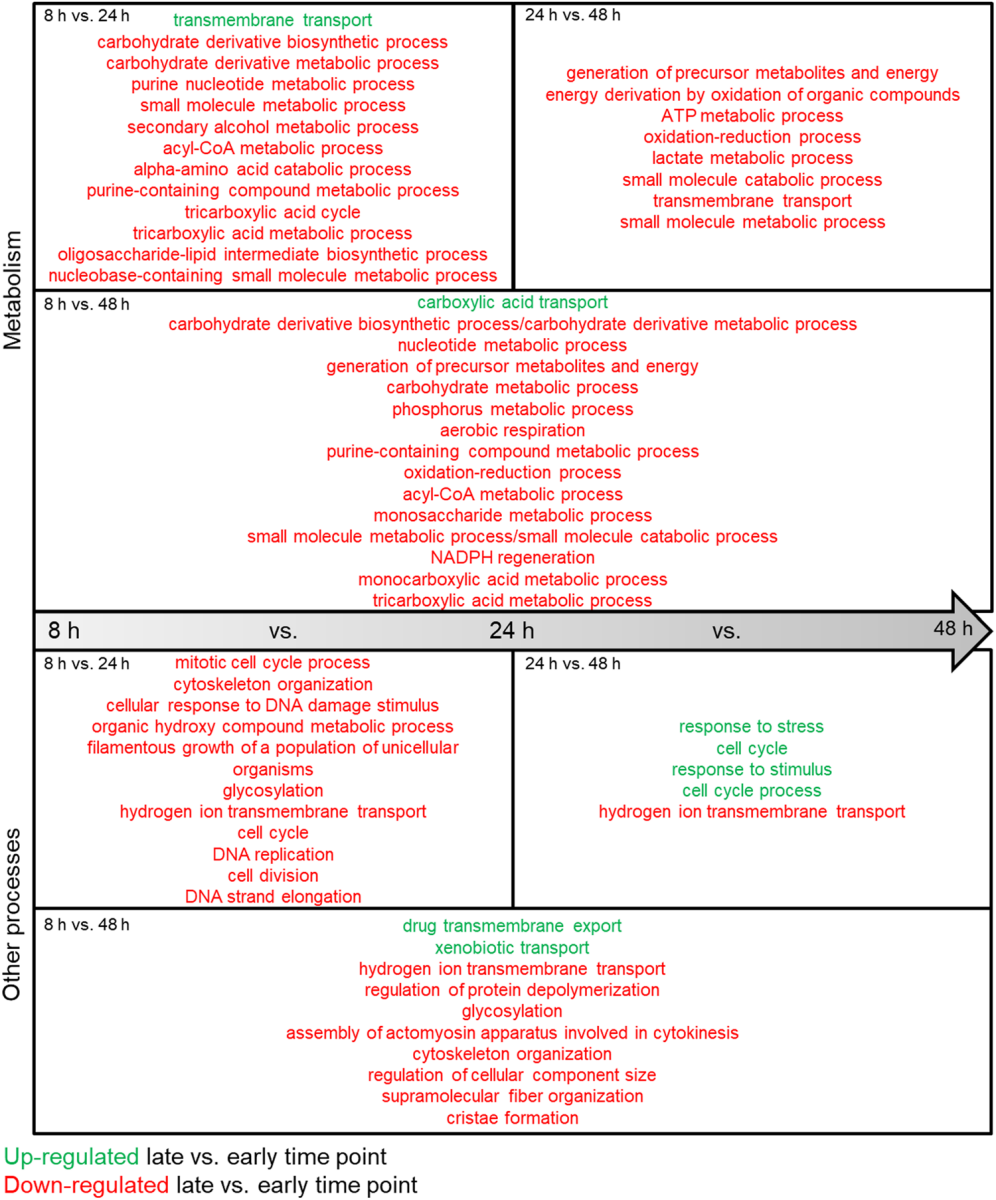
Transcriptional responses in aging biofilms affect various metabolic processes. Differentially regulated genes (log_2_FC ± 1; *p* < 0.05) in late biofilms were compared to earlier time points (24 vs. 8 h; 48 vs. 8 h; 48 vs. 24 h) and GO terms for molecular processes were each summarized using REVIGO with a cutoff of 0.5 dispensability. Metabolism-related processes were separated from others. Up-regulated terms are shown in green, and down-regulated terms are in red. Source data are provided as a Source Data file.

The transcriptional analysis compared aged (24 and 48 h) vs. younger (8 h) biofilms and revealed that gene expression decreases significantly over time for numerous metabolic and other cellular processes (Fig. 1). Down-regulated processes included carbohydrate, purine, amino acid, and energy metabolism pointing towards an increased population of metabolically inactive cells. Besides metabolism, genes for cell cycle and division, DNA replication, cytoskeleton organization, and filamentous growth were transcriptionally repressed during biofilm aging, suggesting slowed or even halted proliferation. Indeed, 48 h biofilms had significantly up-regulated stress response genes indicating increasingly restricted conditions. Genes encoding nutrient transporters were among the few up-regulated terms in older biofilms, demonstrating the growing demand to acquire available nutrients.

**Fig. 2:**
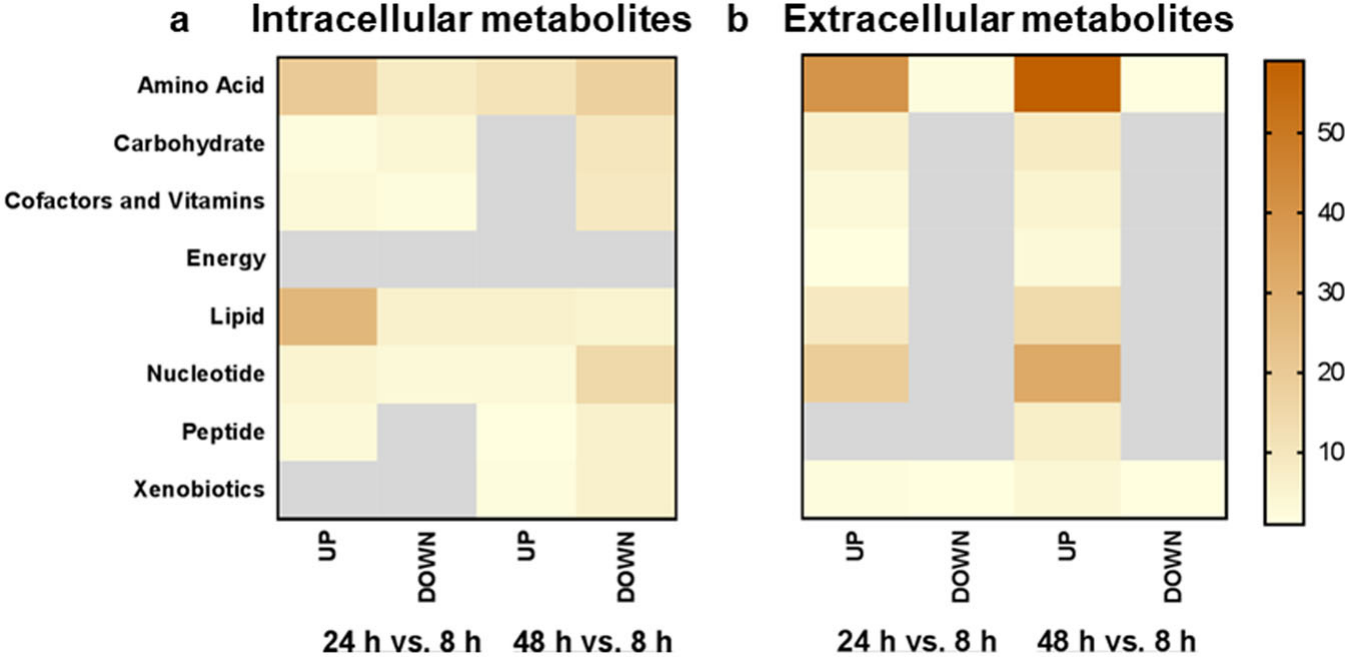
Intracellular and extracellular metabolomics in aging biofilms. Differentially abundant metabolites from each metabolite family were counted and plotted as a heat map. The extent of the metabolic changes is color-coded with darker fillings for bigger changes. The scale bar indicates the metabolite counts within one metabolite family. No significant changes are marked in gray. Cutoff *p* < 0.05 and log_2_FC ± 1. Source data are provided as a Source Data file. **a** The intracellular metabolite content increased after 24 h, but after 48 h of biofilm formation, many metabolites decreased significantly. The largest decline was observed for the nitrogenous substrates (amino acids and nucleotides). **b** Many metabolites accumulated in the extracellular metabolome in mature biofilms as amino acids and nucleotides were most abundant at the late time point.

As biofilm maturation vastly changed the gene expression of metabolism-related genes, we investigated the global metabolome during the time course with an untargeted approach. Overall, we were able to detect 547 metabolites intracellularly and 270 metabolites in the biofilm medium. Comparative analyses of different time points of biofilm formation showed a peak in amino acid and lipid intermediates at 24 h (Fig. 2a). Notably, nitrogen-containing compounds such as amino acids and nucleotides were strongly reduced in 48 h biofilms, which reflects the progressive starvation and the dormant state of mature biofilm cells. The composition of the spent biofilm medium provided information on nutrient uptake and excretion of cellular end products. Extracellular metabolites can originate from the residual components of the culture medium, lysates of dead cells, or actively excreted substrates. The extracellular metabolome was highly enriched in all metabolite classes at later stages, an outcome that correlates with the progressive accumulation of dead cells during biofilm aging (Fig. 2b). The enrichment of extracellular amino acid compounds at 48 h was mainly due to the release of acetylated amino acid derivatives, polyamines and sulfonated amino acids (Supplementary Data File 2). The latter class includes intermediates of cysteine and taurine metabolism that were depleted intracellularly while enriched in the extracellular fraction, implying directed export. As proteins and extracellular DNA are major components of the extracellular biofilm matrix (ECM)^21^, the observed increase in amino acids and nucleotides could partially originate from matrix production. A central carbohydrate component of the *C. albicans* ECM is *β*-1,3-glucan, but we could not detect this polymer. The most extracellularly enriched carbohydrate during biofilm maturation was trehalose.

**Fig. 3:**
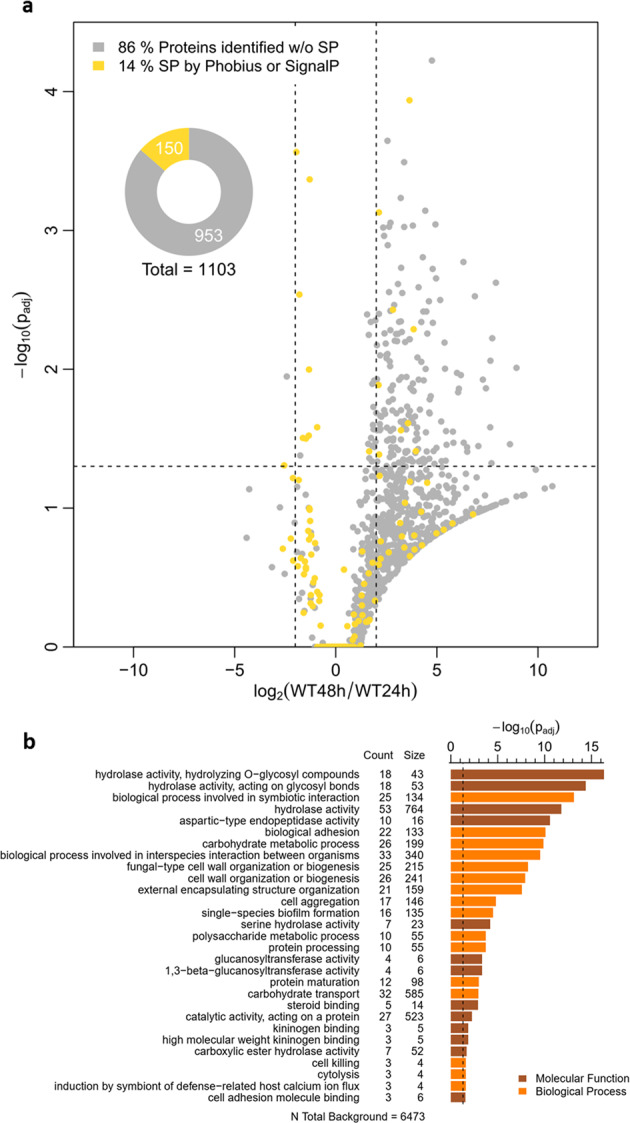
Secretome analysis of mature *C. albicans* biofilm media. **a** Volcano plot illustrating the log_2_ ratio of secreted proteins in 48 and 24 h biofilms to the corresponding *p*_adj_ value (−log_10_). Dashed lines symbolize the cutoff (*p* < 0.05 and log_2_FC ± 2). A total of 1103 different proteins were identified in all biofilms examined, with 801 proteins detected at 24 h and 1067 proteins detected at 48 h. Only 14% contained a signal peptide (SP) for directed secretion. Both the fraction with and without SP showed higher protein quality after 48 h. **b** GO term analysis of all detected proteins containing an SP. Counts represent observed protein abundance compared to the number of proteins assigned to the term (size). Results for molecular function and biological process were each summarized using REVIGO with a cutoff of 0.5 dispensability. Source data are provided as a Source Data file.

In response to starvation, *C. albicans* typically secretes hydrolytic enzymes to acquire nutrients from macromolecules^22^. To investigate nutrient acquisition by biofilm cells, a secretome analysis of spent biofilm media was performed. In total, 1103 proteins were identified, with 801 present in the biofilm supernatant after 24 h and 1067 after 48 h, indicating a progressive protein release during aging (Fig. 3a). In silico analysis identified signal peptides (SP) in 150 proteins (14%), which are supposed to be secreted from the cell or inserted into cellular membranes via the conventional pathway. Gene ontology (GO) term analysis of the secretome core set of SP-proteins from both time points (Fig. 3b) showed enrichment in biofilm-associated processes like adhesion, single-species biofilm formation, and interspecies interaction. Furthermore, we noted enhanced cell aggregation and cell wall organization proteins including proteins related to filamentation and structural integrity of biofilms. Carbohydrate transport reflects the cellular need for nutrient acquisition. The evaluation of the biological function confirmed the secretion of hydrolytic enzymes with peptidase, glucosidase, or chitinase function as well as adhesins. Thus, secreted proteins could contribute considerably to metabolic and structural shifts during biofilm maturation. The enrichment of non-SP proteins showed stress-responsive terms (Supplementary Data File 3), which link cell lysis processes and also matched the transcriptome data.

### Transcriptional and metabolic responses of mature *C. albicans* biofilms resemble hypoxic growth conditions

**Fig. 4:**
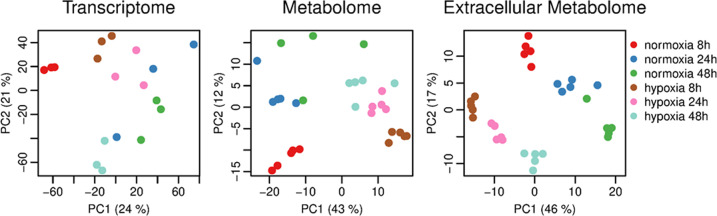
Principal component analyses for transcriptome and metabolome data sets from maturing wild-type biofilms. Gene expression pattern from 8 h normoxic biofilms builds a distinct group, whereas older normoxic biofilms cluster together with hypoxic biofilms from all time points. Intra- and extracellular metabolomes strongly cluster by the oxygen state and show a mostly time-point-independent distribution. Source data are provided as a Source Data file.

**Fig. 5:**
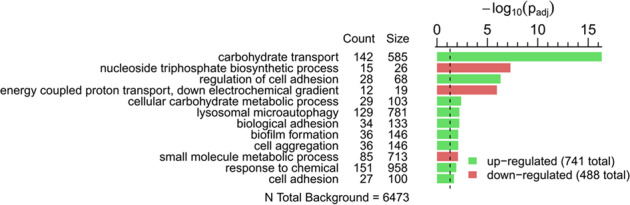
GO term analysis of differentially expressed genes in 8 h wild-type biofilms grown under hypoxia vs. normoxia. Enriched GO terms were determined for hypoxia up-regulated genes (741 total) and hypoxia down-regulated genes (488 total) in contrast to the normoxic counterparts. Count describes the number of measured differentially regulated genes of a process (cluster frequency) and size measures the genomic background frequency. All GO terms were summarized using REVIGO with stringent settings (Cutoff: 0.7 dispensability). Source data are provided as a Source Data file.

**Fig. 6:**
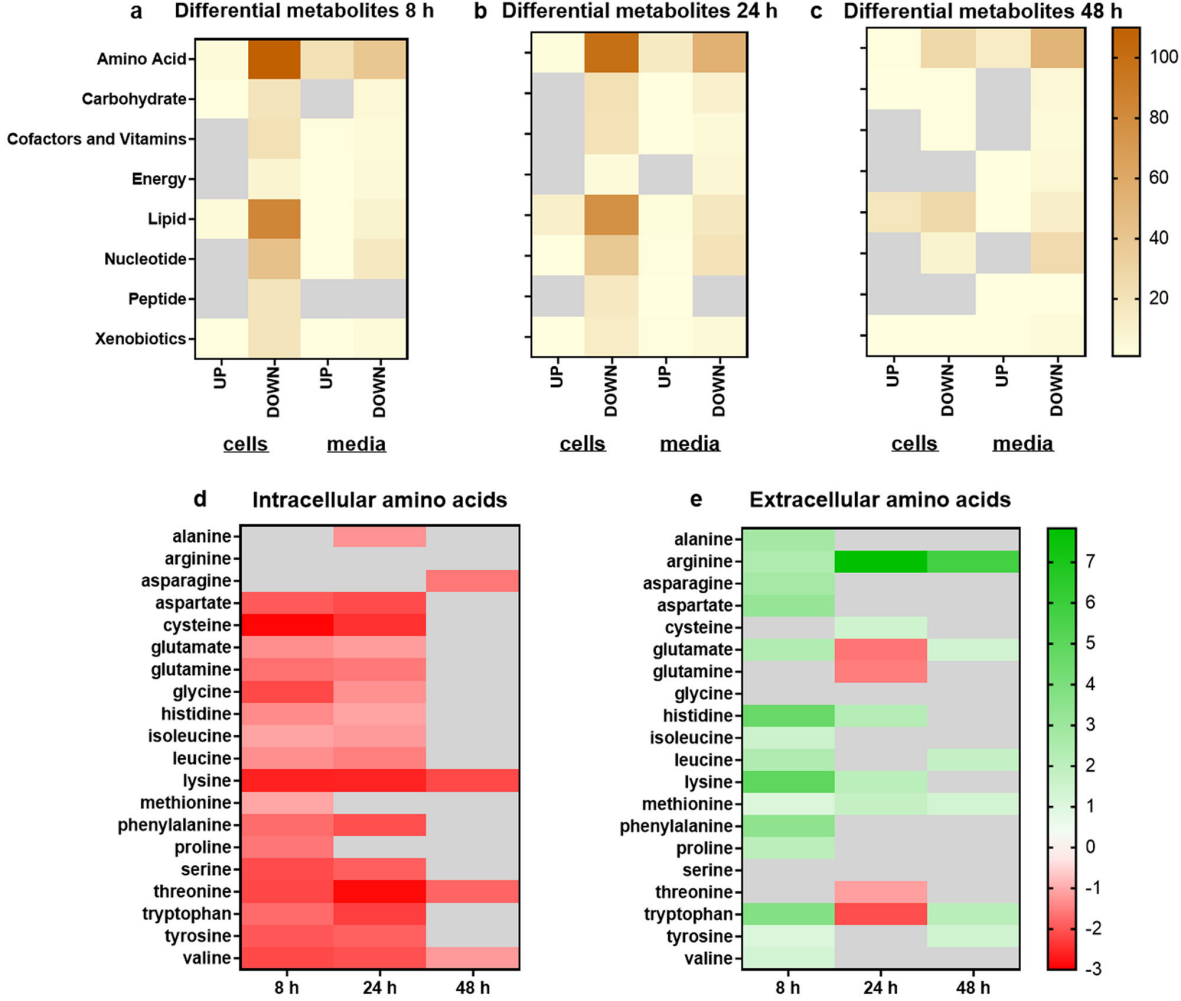
Intracellular and extracellular metabolomics in hypoxic vs. normoxic biofilms. Metabolomes from cells and media from hypoxic biofilms were compared to normoxic biofilms and numbers of significant changes (cutoff *p* < 0.05 and log_2_FC ± 1) were plotted for main metabolic pathways. Numbers of differentially abundant metabolites were analyzed and summarized according to the respective pathways for (**a**) 8 h, (**b**) 24 h, and (**c**) 48 h biofilms. Significant changes (log_2_FC) in the abundance of intracellular (**d**) and extracellular amino acids (**e**) were plotted. Source data are provided as a Source Data file.

Metabolic adaptation during biofilm maturation affected oxygen-demanding processes, such as aerobic respiration. To gain more insight, normoxic biofilms were compared to hypoxic counterparts at early and late stages. Media for oxygen-depleted settings was pre-incubated in a hypoxic chamber to reduce oxygen levels and all biofilms were grown in sealed six-well plates. Interestingly, the projections of the transcriptional and metabolic data with respect to the time course and oxygen levels showed a distinct grouping pattern (Fig. 4). In the principal component analysis (PCA) of the transcriptome only 8 h normoxic biofilms separated from older normoxic and hypoxic biofilms, whereas the latter grouped among themselves. This finding shows a transcriptional response to progressive oxygen limitation in mature biofilms independent of initial oxygen supply. In stark contrast, both extracellular and intracellular metabolomes grouped according to their initial oxygen supply in normoxic and hypoxic biofilm samples, and they split into smaller subgroups reflecting single time points. This data indicates a bigger impact of the initial oxygen availability on the metabolic composition than the time point of biofilm maturation. Since major differences occurred at 8 h of biofilm formation, the early time point was selected for further analyses. As expected, most down-regulated processes, ATP metabolism and mitochondrial electron transport, reflected the oxygen deprivation (Fig. 5). Gene expression of biofilm-related processes was higher in hypoxic biofilms, which could result from faster biofilm growth under normoxia, where transcription of biofilm-related genes had already flattened at the 8 h time point (Fig. 5). Other processes that were differentially regulated under hypoxia included induced autophagy and carbohydrate transport and negatively regulated amino acid catabolic process via the Ehrlich pathway (Fig. 5). In early biofilms, the repressed energy metabolism under hypoxia was also reflected in the cellular metabolome, where only 12 metabolites were enriched and 322 metabolites from all metabolic pathways were substantially less abundant compared to the normoxic biofilms (Fig. 6a). The most reduced metabolites comprise of amino acids, lipids and TCA cycle intermediates, which reflects the impaired mitochondrial metabolism and rearrangements in the cellular lipidome. Although the intracellular and extracellular metabolomes formed distinct clusters in the PCA (Fig. 4), only a small number of metabolites, especially referring to amino acids and nucleotides, differed in the hypoxic spent medium. The temporal trends for the intracellular and extracellular metabolome were similar: in both cases, the absolute number of different abundant metabolites decreased (Fig. 6b, c). The lowest number of differential metabolites was detected after 48 h, indicating increasing similarity between the two biofilm conditions. The remaining differences during the late phase included intracellular changes in amino acids and lipid metabolites, as well as a strong decrease in extracellular nitrogen derivatives. The intracellular levels of proteinogenic amino acids were relatively low except for arginine (Fig. 6d). Simultaneously, most extracellular amino acids were retained longer in the medium under hypoxia (Fig. 6e), but at late time points amino acid levels were similar to the normoxic control.

Multi-omics analysis revealed differences in metabolic adaptations over time: although energy metabolism was generally reduced under hypoxia compared with normoxia, genes, as well as some associated metabolic intermediates of glycolysis and TCA metabolism, were up-regulated at late compared with early hypoxic time points (Supplementary Fig. 1b, d). In contrast, these central metabolic pathways were shut down in counterparts grown under normoxia (Supplementary Fig. 1a, c). We, therefore, conclude that growth under hypoxic conditions requires a long period of adaptation during which central metabolic pathways are restructured to compensate for the lack of cellular respiration.

### Stp2 mediates amino acid uptake during biofilm formation with a wide-ranging impact on metabolic homeostasis

**Fig. 7:**
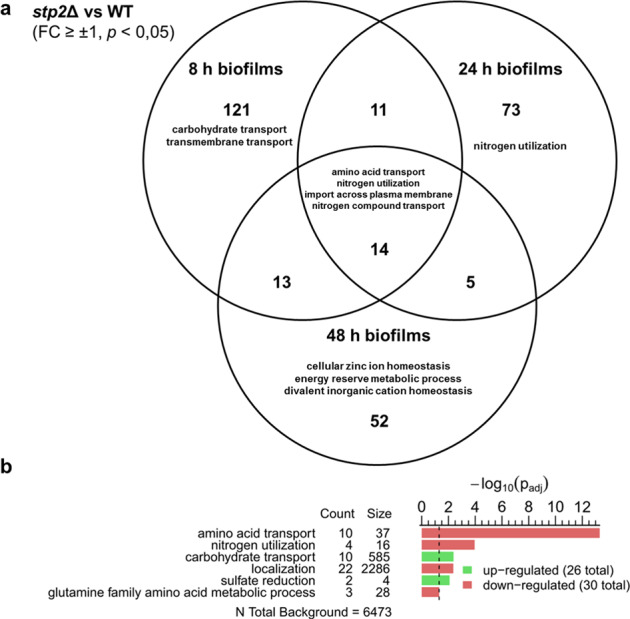
Transcriptome analyses in *stp2*Δ biofilms compared to the wild type under normoxia. Transcriptomes of *stp2*Δ and wild-type biofilms grown for 8, 24, and 48 h under normoxic conditions and differentially expressed genes (*p* < 0.05 and log_2_FC ± 1) were analyzed for GO term enrichments and summarized with REVIGO (dispensability < 0.5 and *p* < 0.05 were displayed). Source data are provided as a Source Data file. **a** VENN diagram of time point-specific effects shows the biggest effects for *stp2*Δ at early time points. **b** Time point-independent (time point as a confounding variable) differences were analyzed for genotype-specific effects during biofilm formation.

The characterization of wild-type *C. albicans* biofilm maturation revealed that transmembrane transporter genes and stress response were the few positively enriched terms in the transcriptome over time, whereas many metabolic and energy-preserving processes were down-regulated (Fig. 1). Intermediates of amino acid biosynthesis pathways were found to be enriched in biofilm cells and medium during maturation. To address the importance of amino acid uptake in mature biofilms, we studied in detail the processes that accompany biofilm formation of the *stp2*Δ mutant, which is impaired in this function^17,19^. PCA of wild-type and *stp2*Δ omics data sets revealed clustering with respect to sampling time points and oxygen levels with less influence on the genotypes (Supplementary Fig. 2). Transcriptomes from *stp2*Δ and wild-type normoxic biofilms were compared at each time point (8, 24 and 48 h). As expected, most affected GO terms in the time point-independent core set belonged to amino acids transporters and nitrogen compound import and utilization (Fig. 7a). At single time points, *stp2*Δ biofilms had increased expression of genes for carbohydrate transport at 8 h and for zinc ion homeostasis and energy reserve metabolism at 48 h. These processes point towards a broader function of Stp2 besides facilitating amino acid uptake. The transcriptome data was adjusted to time to elucidate genotype-related effects, with GO term analysis identifying glutamine metabolism and sulfate reduction found in addition to the core set (Fig. 7b). The up-regulation of sulfate assimilation in the *stp2*Δ background hints toward the acquisition of sulfate for the biosynthesis of sulfur-containing amino acids.

Young *stp2*Δ biofilms (8 h) showed increased expression of *HGT* genes that belong to the family of glucose transporters^23^ (Fig. 7, Supplementary Data File 1 and Supplementary Table 1). In *Saccharomyces cerevisiae*, members of this family were shown to transport quinine in addition to hexoses and that expression of hexose transporters can be chemically induced^24^. Quinine has been used to inhibit glucose transport in fungi and *S. cerevisiae stp2*Δ mutant showed high sensitivity to this agent^24^. Interestingly, a combination of quinine and bicarbonate blocked amino acid and sulfate transport in *C. albicans*^25^. We examined the effect of quinine on the growth of *C. albicans* wild type, *stp1*Δ, *stp2*Δ, and the *stp1*Δ*stp2*Δ double mutant strains. While the addition of quinine or bicarbonate alone did not affect the growth of the tested *C. albicans* strains (Supplementary Fig. 4), the combination of both chemicals resulted in a mild growth defect of the wild-type and the *stp1*Δ mutant and a severely impaired growth of the *stp2*Δ mutant. Both the *STP2* overexpression mutant and to a lesser extent the *STP2* revertant complemented wild-type growth. Interestingly, the *stp1*Δ*stp2*Δ double mutant showed less severe growth defect compared to the *stp2*Δ strain. Thus, deletion of *STP1* in the *stp2*Δ mutant background alleviated the altered nutrient transport.

**Fig. 8:**
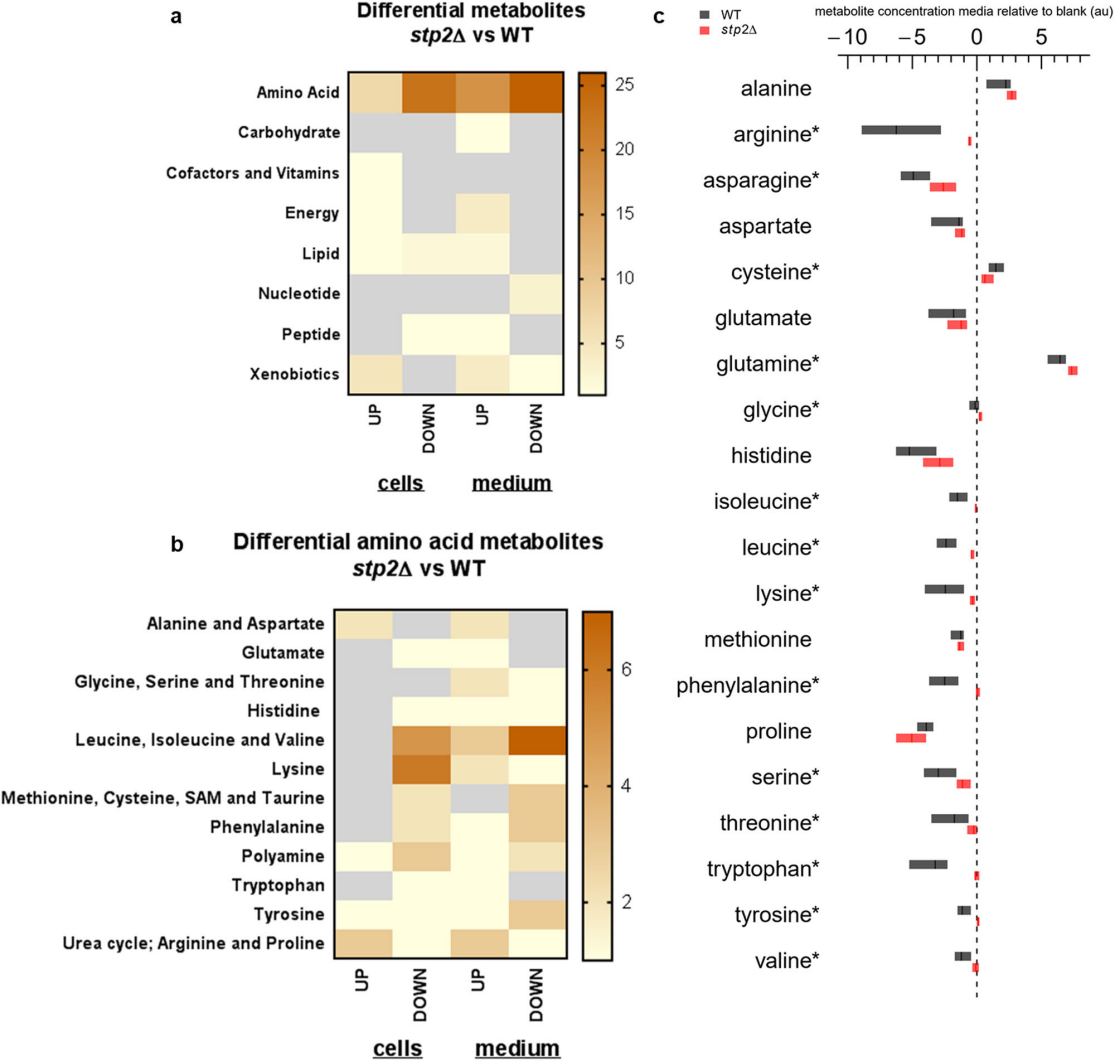
Comparative intracellular and extracellular metabolomics of *stp2*Δ mutant vs. wild-type biofilms. Metabolomes from cells and media were analyzed for significant metabolic changes in *stp2*Δ mutant and wild-type biofilms. Analyzed contrasts were time point-adjusted. Numbers of metabolites with significant changes (Cutoff *p* < 0.05 and log_2_FC ± 1) were plotted for main metabolic families (**a**) and amino acids subgroups (**b**). Metabolite data were time point-adjusted (time point as a confounding variable) before comparative analysis. **c** Box plot diagram shows relative abundance (log_2_FC, median, upper and lower quartiles) of extracellular amino acids from wild-type (black) and *stp2*Δ (red) media, which were normalized to the respective amino acid concentrations from RPMI blank medium. Statistical differences between strains were calculated using the Student’s *t*-test and plotted next to the corresponding amino acid (* for *p* < 0.05). Source data are provided as a Source Data file.

Metabolic comparisons of biofilm cells and supernatants for genotype-specific effects revealed enrichment for amino acids, whereas all other metabolite classes were only slightly affected (Fig. 8a). Intermediates of branched-chain amino acids (BCAA), lysine, and polyamine biosynthesis pathways were most reduced in *stp2*Δ mutant cells. On the other hand, metabolites related to aspartate/asparagine metabolism and urea cycle were increased in the *stp2*Δ biofilm cells (Fig. 8b and Supplementary Fig. 3a). Combined KEGG pathway analysis was used to link transcriptome and metabolome data sets (Supplementary Fig. 3). Although BCAA biosynthesis was transcriptionally up-regulated early, amino acids were enriched only at late time points (Supplementary Fig. 3b). This late biosynthesis could be due to lack of direct precursor intermediates, found at low levels at all time points. In addition, the transaminase gene, which encodes the final enzyme in the biosynthesis of BCAA, was transcriptionally down-regulated. A contrasting example is the citrulline biosynthetic pathway, in which citrulline, arginine, and ornithine were enriched in the intracellular metabolome of *stp2*Δ biofilms (Fig. 8b and Supplementary Fig. 3a). In particular, arginine appeared to be retained for protein biosynthesis, thus downstream metabolic pathways were transcriptionally suppressed, including the *CAR1* arginine transaminase and *CAR2* ornithine transaminase genes. Finally, the subsequent biosynthesis of the polyamines putrescine, spermidine, and spermine were lower in the metabolome of *stp*2Δ biofilms, and the formation and secretion of urea were also reduced (Supplementary Fig. 3c).

The extracellular amino acid composition was analyzed in time course to monitor amino acid uptake for both strains under normoxic conditions (Fig. 8c and Supplementary Table 2). We found an Stp2-mediated uptake for the following amino acids: arginine, asparagine, isoleucine, leucine, lysine, phenylalanine, serine, threonine, tryptophan, tyrosine, and valine. Interestingly, when we compared these amino acids to the published set of amino acids that induce Stp2 processing and activation^26^, we found only five common metabolites: arginine, asparagine, lysine, serine, and threonine (Supplementary Table 2). Proline was the only amino acid with increased import by *stp2*Δ cells compared to the wild type. Alanine, cysteine, and glutamine accumulated in the spent medium over time. Many other products of amino acid metabolism were enriched in the biofilm supernatant (Fig. 8, Supplementary Data File 2), including acetylated amino acids and tyrosol, a secreted byproduct of tyrosine metabolism. Tyrosol, a quorum-sensing molecule that supports hyphal formation^27^, was enriched in wild-type biofilm medium and was significantly lower in the *stp2*Δ counterpart.

*STP2* deletion mutants are severely impaired in amino acid uptake and compensatory mechanisms in nutrient acquisition become increasingly important during the time course of biofilm formation. In this context, the secretion of hydrolytic enzymes that break extracellular macromolecular nutrients, such as polypeptides and lipids, could provide additional nutrient sources. Secretomes of the *stp2*Δ biofilms were compared to the wild type and revealed a qualitatively reduced protein amount in *stp2*Δ to 63% at 24 h and 77% at 48 h, respectively. Consequently, more proteins were differentially enriched in wild-type secretomes (Supplementary Fig. 5a, b). A core set of genotype-specific secreted proteins from both time points was defined (Supplementary Table 3). The *stp2*Δ-specific biofilm secretome contained two hydrolyzing enzymes acting in the acquisition of fatty acids from lysophosphatidylcholine by the phospholipase B (Plb1), and peptides from proteins by the secreted aspartyl protease 2 (Sap2). The wild-type-specific secretome also contained enzymes for nutrient acquisition like the carboxypeptidase Cpy2 and the glucoamylase Gca2. Other wild-type-related proteins were connected to the cell wall assembly, such as glucosidases and mannosyltransferases. The last observation may indicate the role of Stp2 in the protein composition of the cell wall of mature biofilms. The extracellular presence of the enzyme adenylate kinase Adk1 can be used as a marker of cell lysis^28^ and we noted constantly increasing Adk1 abundance in the wild-type secretome (Supplementary Data File 3). The enzyme abundance was lower, albeit not significantly, in the *stp2*Δ secretome compared to the wild type. Nevertheless, the data indicated progressive cell lysis, especially in wild-type biofilms.

## Discussion

*C. albicans* is a common colonizer of the human host, where it grows mainly in multilayered biofilms. Superficial mucosal infections are caused by biofilms from which dispersal cells can detach and thus spread systemically. Living in these complex structures protects the fungus from external attack during an immune response or antifungal treatment^29,30^.

Embedded cells have limited access to preferential nutrients and oxygen due to the biofilm compartmentalization and structure. During biofilm maturation, specialized sub-populations differentiate to adapt to stress conditions like nutrient deficiency^31,32^. Amino acid homeostasis is critical for cellular growth and maintenance and this process includes three main mechanisms: amino acid uptake, de novo synthesis, and recycling by autophagy^33^. Previous studies showed a link between transcriptional regulation of amino acid utilization genes and *C. albicans* biofilm formation^15,18–20^, but the importance of active amino acid uptake and its metabolic consequences have not been addressed. Using a comprehensive multi-omics data set, wild-type normoxic biofilms were compared to hypoxic conditions and an *stp2*Δ mutant with impaired amino acid utilization to examine the effects of progressive oxygen and nutrient limitation, respectively. We deliberately chose defined, static biofilm conditions to allow for direct data comparison, even though these conditions cannot fully represent clinical biofilms.

Our transcriptome data showed decreasing expression of genes involved in the metabolism of carbohydrates, amino acids, and nucleotides, as well as energy production via the TCA cycle and respiration during *C. albicans* biofilm maturation under normoxic conditions. Furthermore, we noted early up-regulation of glycolysis, amino acid metabolism, and lipid biosynthesis, whereas at later time points the utilization of alternative nutrients was increased, well in line with previous observations^14,34^. Transcriptional regulation of metabolic genes resulted in changes in the metabolome, where intracellular amino acid and lipid intermediates peaked at 24 h and dropped later, indicating early active mitochondrial processes and overall reduced metabolic rates during aging. Similar observations were made by Zhu et al. who showed that metabolites belonging to the TCA cycle, amino acids, and carbohydrate intermediates were predominant in the middle phase of biofilm formation^11^.

At later stages, we observed a dramatic decrease in gene expression of several metabolic pathways, but with a particular increase in amino acid permease genes that are likely required to maintain intracellular amino acid homeostasis. The up-regulation of amino acid and peptide transporters seems to be a conserved feature of microbial biofilms reported for gram-negative and gram-positive bacteria, yeasts, and filamentous fungi^5,10,16,34–37^. Interestingly, previous work by Traven et al. showed that genes for amino acid and fermentative metabolism were highly expressed in the upper cell layers of *S. cerevisiae* colonies compared to the lower structures. The latter show particularly high expression of mitochondrial respiration genes, which is indicative of slowed metabolism in the bottom layers and active growth in the upper biofilm layers^38^.

Restricted gas diffusion and oxygen-deficient environments are also considered common features of bacterial and fungal biofilms^29,35,39^. For human pathogens, the formation of hypoxic niches in biofilms is of particular therapeutic importance because, as exemplified by *Aspergillus fumigatus*, the reduced metabolic state is associated with increased antifungal resistance^29^. We demonstrated an increasing oxygen deficiency of the initially normoxic biofilms at the level of gene expression, as the profiles approached the hypoxia-grown biofilms at later time points. Transcriptional down-regulation of energy production was reflected in greatly reduced levels of intracellular metabolites and consequently lower secretion of nitrogenous metabolites into the medium. The low metabolic rate led to the induction of autophagy genes, which is a general response to starvation for various essential nutrients, where cytosolic proteins are targeted to the vacuole for bulk degradation and recycling of amino acids^33,40^. Further metabolic compensation was achieved by transcriptional induction of nutrient import, which resembles enhanced transcription of amino acid permease genes in planktonic cells grown under hypoxia^41^. The metabolic profiles were more temporally stable than the transcriptional fingerprint and separated normoxic and hypoxic metabolic patterns at each time point. Hypoxic growth requires rearrangements of metabolic pathways in *C. albicans*, such as increased transcription of glycolytic genes is necessary for adaptation to oxygen deficiency^10^. In a similar manner, glycolysis and, to some extent, the TCA cycle were transcriptionally up-regulated in late hypoxic biofilms. Metabolic responses to sustained hypoxia resulted in the degradation of TCA cycle intermediates and lipids in planktonic cells^41^. However, we observed an increase in lipids and TCA metabolites at late time points, suggesting a biofilm-specific effect.

Wild-type biofilms underwent an extensive restructuring of amino acid metabolism, and an *STP2* deletion mutant deficient in amino acid uptake was selected for further analysis. In a recent study, we showed that Stp2 is involved in adherence and early biofilm formation and gene knockout had a positive effect on biofilm longevity^17^, but the underlying processes have not been investigated. In the current study, transcriptome analyses revealed that along the expected expression control of amino acid permease genes, Stp2 had an overall impact on cellular nitrogen metabolism at all time points of biofilm formation.

Impaired amino acid uptake leads to intracellular starvation responses. The starvation status of a cell can be tested by adding quinine, which artificially creates further nutrient stress leading to increased sensitivity in pre-starved cells. In yeast, quinine both disrupts a high-affinity tryptophan/tyrosine transporter and inhibits glucose uptake^24,42^. For *C. albicans* and other fungi, the combination of quinine and sodium bicarbonate was described to induce synergistic growth inhibition^25^. Upon quinine addition, an *S. cerevisiae stp1*Δ mutant was more resistant than the wild type, whereas deletion of Sc*STP2* had the opposite effect^24^. Similarly, combinatory treatment as described above resulted in a near-lethal outcome for the *C. albicans stp2*Δ mutant, whereas an *stp1*Δ mutant showed wild-type growth. The *stp1*Δ*stp2*Δ double mutant had improved growth compared to the *stp2*Δ strain. This result suggests opposing functions for the two *C. albicans* Stp paralogs under severe nutrient stress, with Stp2 being a key starvation factor to counteract the amino acid imbalance.

In order to specify the Stp2-mediated amino acid uptake, extracellular metabolomes were compared to a blank medium and revealed retention of 11 proteinogenic amino acids in the *stp2*Δ spent medium. An exception was proline with increased uptake in the *stp2*Δ background, which may indicate compensation for the generally restricted amino acid uptake or a slightly repressive function of Stp2 in proline uptake. Sensing of extracellular amino acids via the SPS signaling pathway leads to activation of Stp2, and only a subset of eight amino acids can trigger the cascade^19,26^. Surprisingly, only a partial overlap was found between the amino acids that induce Stp2 processing and the ones with Stp2-regulated uptake. In conclusion, although the recognition and uptake of extracellular amino acids are associated with the SPS system, they are not congruent.

The intracellular amino acid abundance of the *stp2*Δ mutant was also reduced and mainly affected branched-chain amino acids and lysine intermediates whose import was Stp2-dependent. Often, direct metabolic precursors or degradation products of these proteinogenic amino acids were depleted, probably to maintain the cellular pool for protein biosynthesis. For instance, conversion of arginine to polyamines was markedly reduced in *stp2*Δ biofilms by down-regulation of arginine and ornithine transaminase genes leading to intracellular accumulation of arginine and ornithine, and reduction of putrescine, spermine, and spermidine. A correlation between polyamine abundance and biofilm development has already been described, such that putrescine prototrophy and extracellular putrescine supplementation contribute to the switch of yeast to hyphae and thus promote biofilm formation^11,43^. The Ptk2 protein kinase is required for efficient polyamine uptake and we observed that *PTK2* expression was increased during the maturation of wild-type biofilms. Similarly, *PTK2* was shown to be transcriptionally up-regulated in biofilms compared to planktonic growth^15^. As a result, both polyamine uptake and intracellular levels support biofilm formation and an imbalance could contribute to the delay of *stp2*Δ biofilm development.

Another way in which metabolism regulates biofilm production is through the secretion of quorum sensing molecules, which can have enhancing or repressive effects^44^. One representative is tyrosol, which is formed by tyrosine degradation and is itself a secreted quorum sensing molecule that acts as a farnesol antagonist, promoting germination and early biofilm formation^27,44,45^. Tyrosol is produced from tyrosine via the Ehrlich-Fusel oil pathway by transaminase, decarboxylase, and alcohol dehydrogenase reaction steps^46^. The production of aromatic alcohols from aromatic amino acids is conserved in *S. cerevisiae* and *C. albicans*^47^. Tyrosol accumulated intracellularly and extracellularly during wild-type biofilm maturation but was suppressed due to the absence of *STP2*. The latter can be explained by the impaired uptake of extracellular tyrosine in *stp2*Δ cells and furthermore by a strongly reduced expression of the decarboxylase gene *ARO10* (35). The formation of the amino acid-derived quorum sensing molecule tyrosol links metabolism to biofilm development and limitations within the pathway correlated with delayed filament formation^17^.

Although we reported severe metabolic limitations at various levels after the loss of *STP2*, the mutant could form structurally intact biofilms at later time points^17^. Several compensatory mechanisms have been identified, including enhanced amino acid biosynthesis. Interestingly, the deletion of *STP2* altered transcription of non-amino acid transporters, including nucleotide and glucose importers with carbohydrate transport being a significantly up-regulated GO term in *stp2*Δ cells compared to the wild type. Recently, biofilm growth under elevated CO_2_ conditions was connected to enhanced glucose uptake via the same set of glucose importers^16^.

A second metabolic adaptation strategy is the increased expression of zinc uptake genes in late *stp2*Δ biofilms. Zinc acquisition and biofilm formation are linked by the transcription factor Csr1, which controls both processes^48,49^. Gene expression for zinc, iron and amino acid uptake was found to be simultaneously up-regulated in dispersal cells, thus playing an important role in the final stage of biofilm development^50^. Given the ability to scavenge zinc more efficiently, the *stp2*Δ mutant could enhance trace metal metabolism, possibly contributing to prolonged longevity.

The third mechanism of starvation response is the secretion of hydrolytic enzymes to access macromolecular nutrients in the environment. Inhibition of these extracellular hydrolases has been used to combat clinical biofilms, indicating their importance in virulence^51,52^. Here, we investigated how the biofilm secretome was affected by *STP2* deletion. First, the significant increase in the amount of secreted proteins in the wild-type medium was particularly striking, likely resulting from early cell lysis compared to the increased longevity of the *stp2*Δ mutant biofilms^17^. A large fraction consisted of cytosolic proteins lacking a predicted SP. Two published studies on the secretome of *C. albicans* found 225 and 446 proteins, respectively, of which about one-third and one-quarter lack a secretion signal^53,54^. In the wild-type secretome, proteins with an SP were frequently involved in either cell wall biogenesis with glucosidase or chitinase activity or were hydrolytic proteins for nutrient acquisition. Both groups belong to non-GPI proteins that are generally predominant in the *C. albicans* secretome^55^. Second, strain-specific proteins were examined along with the wild-type secretome containing an *α*-1,2-mannosyltransferase (Mnn22) and an *α*-glucosidase (Rot2), indicating possible differences in cell wall composition^56,57^. The additional enrichment of the Gca2-glucoamylase may point to Stp2-driven carbohydrate hydrolysis with a possible effect on biofilm matrix formation^58^. To compensate for nutrient depletion, the *stp2*Δ-specific secretome was enriched for a phospholipase B (Plb1) and a secreted aspartyl proteinase (Sap2), which, in addition to their respective lipolytic and proteolytic enzyme functions, are important virulence factors involved in host cell penetration and tissue damage^59,60^.

All in all, with our state-of-the-art multi-omics design we were able to give unique in-depth profiling of *C. albicans* biofilm maturation. We found that the progressive limitation in preferred nutrients and oxygen deprivation resulted in the release of cellular proteins from aging biofilms. By studying an *STP2* deletion mutant we were able to prevent the necessary uptake of a defined set of amino acids. The adaptive nature of the *C. albicans* metabolism facilitated amino acid homeostasis and compensates for the impaired import by activation of intracellular amino acid biosynthesis, carbohydrate import, and secretion of hydrolytic enzymes. Those metabolic rearrangements led to proper biofilm maturation despite starvation stress and even reduced cytolysis during aging. As metabolism is a master regulator in biofilm maturation, interfering with key components of amino acid or sugar uptake could be utilized in combined fungicidal therapy to control fungal biofilm infections.

## Methods

### Strains and cultivation

*C. albicans* strains were routinely passaged on YPD agar (2% peptone, 1% yeast extract, 2% glucose, 1.5% agar) at 30 °C and stored as frozen stocks in YPD medium using Roti®-Store yeast cryovials (Carl Roth GmbH+Co. KG). RPMI with stable glutamine and NaHCO_3_ buffer (product reference FG1215, Merck–Biochrom) was used as induction media for in vitro biofilm and planktonic cultures assays. *C. albicans* strains used in this work are listed in Table 1.

**Table 1:** *Candida albicans* strains in this study.

Name	Genotype	Reference
Wild type SC5314	wild type	^72^
*stp2*Δ	*stp2∆::FRT/stp2∆::FRT/stp2∆::FRT*	^73^
*stp2*Δ+*STP2*	*stp2∆::FRT/stp2∆::FRT/stp2∆::FRT-STP2*	^73^
*STP2*^OE^	*ADH1/adh1::STP2-SAT1*	^74^
*stp1*Δ	*stp1∆::FRT/stp1∆::FRT*	^75^
*stp1*Δ *stp2*Δ	*stp1∆::FRT/stp1∆::FRT* *stp2∆::FRT/stp2∆::FRT/stp2∆::FRT*	^75^

Strains with their genotypes that were used in the study.

### Growth and stress tests

*C. albicans* cells were grown overnight in YPD at 30 °C and washed twice with PBS. Cell density was set to 4 and 5 µl from a 10-fold serial dilution was spotted on selected media and plates were incubated for 2 days at 30 °C. The effect of quinine (quinine hydrochloride dihydrate, Sigma-Aldrich Chemie GmbH, Germany) and bicarbonate (sodium bicarbonate, Sigma-Aldrich Chemie GmbH, Germany) was chosen as cellular stressors as established before^25^.

### Sample preparation for global transcriptomics and metabolomics

Precultures were incubated in 10 ml YPD (overnight, 30 °C, 180 rpm, normoxia). Cells were harvested and split in two for further handling under either normoxic or hypoxic conditions. From this point on the entire experimental procedure, including handling of the medium, was carried out under normoxic (covered by sealing foil) or hypoxic conditions with 1% O_2_ and 0.1% CO_2_ in a hypoxic chamber (SCI-tive, Baker Ruskinn, The Baker Company). Biofilms were induced in 4 ml RPMI medium each well in a six-well plate (Costar®, Corning®) to a density of OD_600 nm_ of 0.5. For each strain and growth condition, six technical replicates were cultured on one well plate. After 90 min of cultivation at 37 °C with shaking (180 rpm), non-adherent cells were rinsed with PBS. Next, fresh, pre-warmed 4 ml RPMI medium was added to the adherent, RPMI-adapted cells. After biofilm formation (37 °C, 180 rpm) for 8 h, 24, or 48 h all six wells of each plate were pooled, centrifuged, and processed as one sample replicate. In addition, a spent biofilm medium was collected for extracellular metabolomic analyses. Biological triplicates were collected for RNA sequencing and five replicates of extracellular and cellular samples were taken for metabolome analysis. All samples were stored at –80 °C until further processing.

### RNA preparation, sequencing, and differential gene expression analysis

#### RNA quantification and qualification

RNA was extracted using RNeasy Plus Universal kit (Qiagen, Hilden, Germany) according to the manufacturer’s protocol. RNA degradation and contamination were monitored on 1% agarose gels. RNA purity was checked using the NanoPhotometer® spectrophotometer (IMPLEN, CA, USA). RNA integrity and quantitation were assessed using the RNA Nano 6000 Assay Kit of the Bioanalyzer 2100 system (Agilent Technologies, CA, USA).

#### Library preparation for transcriptome sequencing

A total amount of 1 µg RNA per sample was used as input material for the RNA sample preparations. Sequencing libraries were generated using NEBNext® UltraTM RNA. Library Prep Kit for Illumina® (NEB, USA) following manufacturer’s recommendations and index codes were added to attribute sequences to each sample. Briefly, mRNA was purified from total RNA using poly-T oligo-attached magnetic beads. Fragmentation was carried out using divalent cations under elevated temperature in NEBNext First Strand Synthesis Reaction Buffer (5X). First-strand cDNA was synthesized using random hexamer primer and M-MuLV Reverse Transcriptase (RNase H-). Second strand cDNA synthesis was subsequently performed using DNA polymerase I and RNase H. Remaining overhangs were converted into blunt ends via exonuclease/polymerase activities. After adenylation of 3’ ends of DNA fragments, NEBNext Adaptor with hairpin loop structure was ligated to prepare for hybridization. In order to select cDNA fragments of preferentially 150–200 bp in length, the library fragments were purified with the AMPure XP system (Beckman Coulter, Beverly, USA). Then 3 µl USER Enzyme (NEB, USA) was used with size-selected, adaptor-ligated cDNA at 37 °C for 15 min followed by 5 min at 95 °C before PCR. Then PCR was performed with Phusion High-Fidelity DNA polymerase, Universal PCR primers, and Index (X) Primer. At last, PCR products were purified (AMPure XP system) and library quality was assessed on the Agilent Bioanalyzer 2100 system.

#### Clustering and sequencing

The clustering of the index-coded samples was performed on a cBot Cluster Generation System using PE Cluster Kit cBot-HS (Illumina) according to the manufacturer’s instructions. After cluster generation, the library preparations were sequenced on an Illumina Novaseq 6000 platform and 150 bp paired-end reads were generated.

#### Bioinformatic and statistical analysis of transcriptome data

FastQC (version 0.11.9) was used to confirm the quality of raw data. Reference genome (*C_albicans*_SC5314_version_A22-s07-m01-r44_chromosomes.fasta) and genome annotation (*C_albicans*_SC5314_version_A22-s07-m01-r44_features.gtf) were downloaded from the *Candida* Genome Database. Paired-end reads were mapped to the reference genome using Bowtie 2 (version 2.3.5.1). Mapped reads were assigned to genomic features using function featureCounts of the R package Rsubread (version 2.2.6). Contrasts between sample groups were calculated using R package DESeq2 (version 1.28.1) and *p*-values were adjusted according to Benjamini–Hochberg. Time-independent fold changes between sample groups were analyzed using models with the time point as a confounding variable.

#### Gene ontology (GO) analysis

Gene lists of contrasts of interest were filtered for significant differences (cut off *p* < 0.05 and log_2_FC ± 1) and analyzed for enriched GO terms regarding the biological process or molecular function using the CGD gene ontology term finder online tool with default settings (*Candida* Genome Database http://www.candidagenome.org/^61^ accessed in September 2021). GO data was summarized by removing redundant terms using REVIGO^62^ with the indicated dispensability cut-off.

#### Multi-omics pathway analysis

The R package path view (version 1.28.1) was used to visualize metabolic and transcriptional fold changes in selected pathways^63^.

### Non-targeted global metabolite profiling

Sample preparation and analysis were carried out as described previously^64^ at Metabolon, Inc. In brief, sample preparation involved protein precipitation and removal with methanol, shaking, and centrifugation. The resulting extracts were profiled on an accurate mass global metabolomics platform consisting of multiple arms differing by chromatography methods and mass spectrometry ionization modes to achieve broad coverage of compounds differing by physiochemical properties such as mass, charge, chromatographic separation, and ionization behavior. The details of this platform have been described previously^65,66^. Metabolites were identified by automated comparison of the ion features in the experimental samples to a reference library of chemical standard entries that included retention time, molecular weight (*m*/*z*), preferred adducts, and in-source fragments as well as associated MS spectra, and were curated by visual inspection for quality control using software developed at Metabolon^64,67^.

#### Statistical analysis of metabolome data

All metabolite raw data were normalized in terms of raw area counts between samples. Subsequently each biochemical was rescaled to set the median equal to 1. Then, missing values were set to the value of the smallest non-missing value for each metabolite. Fold changes between sample groups and p-values were calculated using a two-sided *t*-test. Time-independent fold changes between sample groups were analyzed using linear regression with the time point as a confounding variable. *p*-values were adjusted according to Benjamini–Hochberg. Replicate two of the intracellular metabolome sample “wild type, normoxia, 48 h” was considered a technical outlier due to a 25-fold lower mean metabolite abundance and limited metabolite detection.

### Extracellular proteome

#### In-solution digest

Normoxic biofilms were grown for 24 or 48 h in three six-well plates for each sample in order to collect 50 ml of spent biofilm media. Experiments were performed in biological triplicates. The supernatant of spent liquid culture medium was treated as follows: 1 tablet of Complete Ultra protease inhibitor cocktail (Roche) was added to 50 ml filtered supernatant. The pH was set to 2 by the addition of trifluoroacetic acid (TFA). Proteins were enriched by using solid-phase extraction with Chromabond C4 SPE cartridges (Macherey-Nagel). The silica-bound C4 matrix was first reconstituted with 4 ml acetonitrile (ACN) and conditioned with 4 ml 0.1% TFA in water. Then the sample was loaded. The cartridges were washed using 4 ml 5% methanol (MeOH)/0.1% TFA in water. Proteins were eluted with 4 ml 0.1% TFA in 80/20 ACN/water (v/v). The eluate was evaporated until dry by using a vacuum concentrator. The proteins were resolubilized in 100 µl 50 mM triethylammonium bicarbonate (TEAB) in 50/50 2,2,2-trifluoroethanol (TFE)/water (v/v). Protein thiols were reduced and alkylated for 30 min at 70 °C after the addition of each 4 µl 500 mM TCEP (tris(2-carboxyethyl)phosphine) and 625 mM 2-chloroacetamide (CAA). The samples were further cleaned up by methanol–chloroform–water precipitation using the protocol of Wessel and Flügge^68^. The precipitate was resolubilized in 100 µl 5% TFE/95 mM TEAB in water and digested overnight (18 h) with a Trypsin + LysC mixture (Promega) at a protein to protease ratio of 25:1. Samples were again evaporated in vacuum concentrator, resolubilized in 25 µl of 0.05% TFA in H_2_O/ACN 98/2 (v/v) filtered through Ultrafree-MC 0.2 µm PTFE membrane spin filters (Merck-Millipore). The filtrate was transferred to HPLC vials and injected into the LC–MS/MS instrument. Each sample was measured in triplicate (3 analytical replicates of three biological replicates).

#### LC–MS/MS analysis

LC–MS/MS analysis was performed on an Ultimate 3000 nano RSLC system connected to a QExactive HF mass spectrometer (both Thermo Fisher Scientific, Waltham, MA, USA). Peptide trapping for 5 min on an Acclaim Pep Map 100 column (2 cm × 75 µm, 3 µm) at 5 µl/min was followed by separation on an analytical Acclaim Pep Map RSLC nano column (50 cm × 75 µm, 2 µm). Mobile phase gradient elution of eluent A (0.1% (v/v) formic acid in water) mixed with eluent B (0.1% (v/v) formic acid in 90/10 acetonitrile/water) was performed as follows: 0–5 min at 4% B, 30 min at 7% B, 60 min at 10% B, 100 min at 15% B, 140 min at 25% B, 180 min at 45% B, 200 min at 65% B, 210–215 min at 96% B, 215.1–240 min at 4% B.

Positively charged ions were generated at a spray voltage of 2.2 kV using a stainless steel emitter attached to the Nanospray Flex Ion Source (Thermo Fisher Scientific). The quadrupole/orbitrap instrument was operated in Full MS/data-dependent MS2 (Top15) mode. Precursor ions were monitored at *m*/*z* 300–1500 at a resolution of 120,000 full width at half maximum (FWHM) using a maximum injection time (ITmax) of 120 ms and an automatic gain control (AGC) target of 3 × 10^6^. Precursor ions with a charge state of *z* = 2–5 were filtered at an isolation width of *m*/*z* 1.6 amu for further HCD fragmentation at 30% normalized collision energy (NCE). MS2 ions were scanned at 15,000 FWHM (ITmax = 120 ms, AGC = 2 × 10^5^) using a fixed first mass of *m*/*z* 120 amu. Dynamic exclusion of precursor ions was set to 30 s and the underfill ratio was set to 1.0%. The LC–MS/MS instrument was controlled by Chromeleon 7.2, QExactive HF Tune 2.8, and Xcalibur 4.0 software.

#### Protein database search

Tandem mass spectra were searched against the UniProt database of *Candida albicans* (https://www.uniprot.org/proteomes/UP000000559; 2019/06/16) using Proteome Discoverer (PD) 2.2 (Thermo) and the algorithms of Mascot 2.4.1 (Matrix Science, UK), Sequest HT (version of PD2.2) and MS Amanda 2.0. Two missed cleavages were allowed for the tryptic digestion. The precursor mass tolerance was set to 10 ppm and the fragment mass tolerance was set to 0.02 Da. Modifications were defined as dynamic Met oxidation, and protein N-term acetylation as well as static Cys carbamidomethylation. A strict false discovery rate (FDR) < 1% (peptide and protein level) was required for positive protein hits. If only 1 peptide per protein has been identified the hit was accepted if the Mascot score was >30 or the MS Amanda score was >300 or the Sequest score was >4. The Percolator node of PD2.2 and a reverse decoy database were used for *q*-value validation of spectral matches. Only rank 1 proteins and peptides of the top-scored proteins were counted. Label-free protein quantification was based on the Minora algorithm of PD2.2 using a signal-to-noise ratio >5. Imputation of missing quan values was applied by setting the abundance to 75% of the lowest abundance identified for each sample.

#### Bioinformatic and statistical analysis of proteome data

Fold changes between sample groups and *p*-values were calculated using a two-sided *t*-test. The ratio-adjusted *p*-value was derived from the *p*-value divided by the log_4_ ratio. Webserver versions of SignalP^69^ and Phobius^70^ were used to predict potential signal peptides in sequences of all identified proteins.

## Supplementary information

Supplemental Material

S1 Transcriptomics data set

S2 Metabolomics data set

S3 Secretome data set

## Data Availability

The transcriptomics datasets generated during the current study and analyzed in Figs. 1, 4, 5, 7, S1–3, and Supplementary Table 1 are available in the NCBI Gene Expression Omnibus with the dataset identifier GSE194223. The mass spectrometry proteomics datasets generated and analyzed in Figs. 3, S4, and Supplementary Table 3 during the current study have been deposited to the ProteomeXchange Consortium via the PRIDE^71^ partner repository with the dataset identifier PXD026449. The source data underlying Figs. 1–8, Supplementary Figs. 1–4 and Supplementary Table 2 are provided as Source Data files. The data in Fig. S5 is included in this published article.
