# Supplementary material for: Impaired amino acid uptake leads to global metabolic imbalance of Candida albicans biofilms
Source: NPJ Biofilms Microbiomes. 2022 Oct 13;8:78. doi: 10.1038/s41522-022-00341-9 (PMC9556537; doi:10.1038/s41522-022-00341-9)
Supplement: Supplementary file 1 — Supplemental Material [file 41522_2022_341_MOESM1_ESM.pdf]

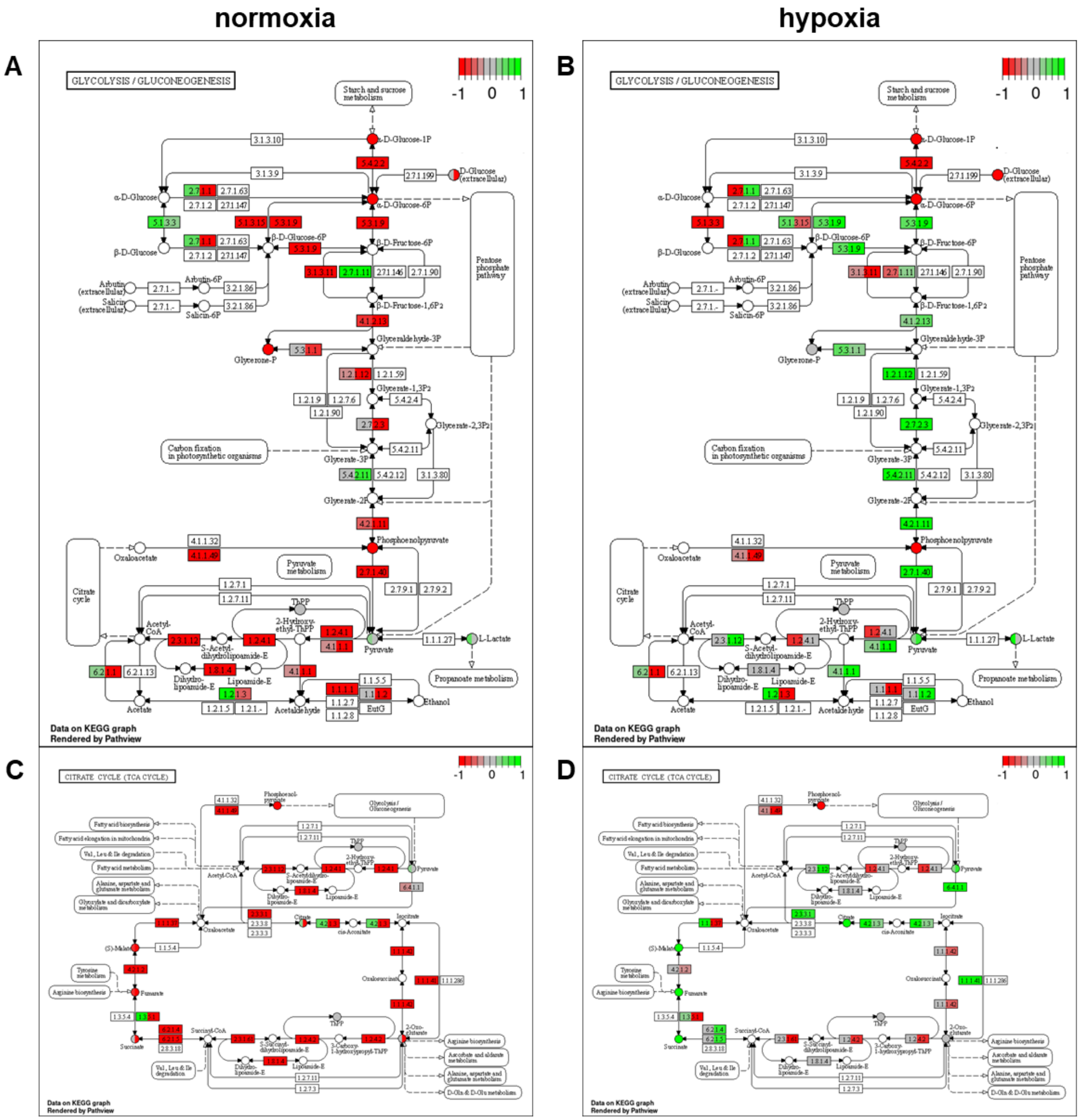

Supplementary Figure 1 Multi-omics analyses of glycolysis and TCA pathways combine transcriptomics and metabolic data sets in the time course of wild-type biofilm maturation under normoxia and hypoxia.

Rectangles represent differential gene expression and circles show differential metabolite abundance in wild type biofilms at 24 h (left filling) and 48 h (right filling) vs. the 8 h time point.

Multi-omics analysis of transcriptional and metabolic changes involved in glycolysis under normoxia (A) and hypoxia (B) and in TCA pathway under normoxia (C) and hypoxia (D).

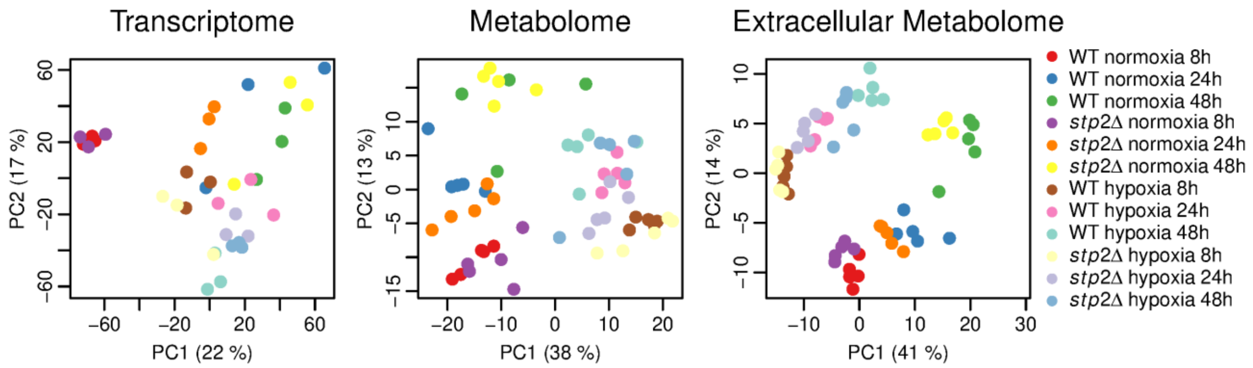

Supplementary Figure 2 Principal component analyses for transcriptome and metabolome data sets from maturing wild-type and *stp2*Δ biofilms

Wild-type and *stp2*Δ-omics datasets revealed clustering with respect to sampling time points and oxygen levels with little influence of their genotypes.

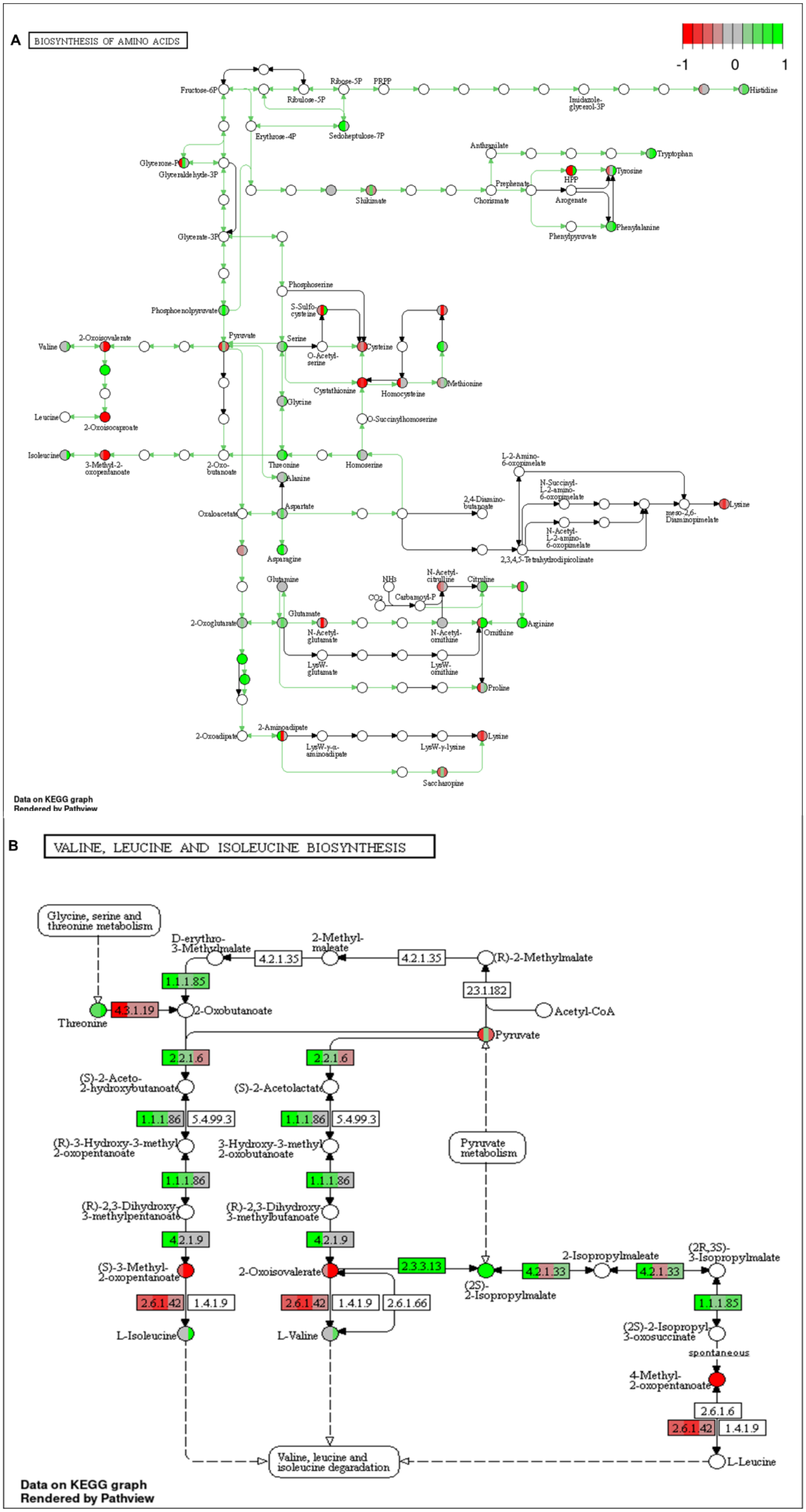
A
BIOSYNTHESIS OF AMINO ACIDS
-1
0
1
Fructose-6P
Ribulose-5P
Ribose-5P
PRPP
Imidazole-glycerol-3P
Histidine
Erythrose-4P
Sedoheptulose-7P
Glycerone-P
Glyceraldehyde-3P
Anthranilate
Tryptophan
HPP
Tyrosine
Prephenate
Shikimate
Chorismate
Arogenate
Phenylpyruvate
Phenylalanine
Glycerate-3P
Phosphoserine
Phosphoenolpyruvate
S-Sulfo-cysteine
Valine
2-Oxoisovalerate
Pyruvate
Serine
Cysteine
O-Acetyl-serine
Cystathionine
Homocysteine
Methionine
Glycine
Leucine
2-Oxoisocaproate
O-Succinylhomoserine
Isoleucine
3-Methyl-2-oxopentanoate
2-Oxo-butanoate
Threonine
Homoserine
Alanine
L-2-Amino-6-oxopimelate
N-Succinyl-L-2-amino-6-oxopimelate
2,4-Diamino-butanoate
Oxaloacetate
Aspartate
Lysine
meso-2,6-Diaminopimelate
N-Acetyl-L-2-amino-6-oxopimelate
Asparagine
2,3,4,5-Tetrahydrodipicolinate
Glutamine
NH3
N-Acetyl-citrulline
Citrulline
CO2
Carbamoyl-P
2-Oxoglutarate
Glutamate
N-Acetyl-glutamate
N-Acetyl-ornithine
Ornithine
Arginine
LysW-glutamate
LysW-ornithine
Proline
2-Aminoadipate
2-Oxoadipate
LysW-γ-α-aminoadipate
LysW-γ-lysine
Lysine
Saccharopine
Data on KEGG graph
Rendered by Pathview
B
VALINE, LEUCINE AND ISOLEUCINE BIOSYNTHESIS
Glycine, serine and threonine metabolism
D-erythro-3-Methylmalate
4.2.1.35
2-Methyl-maleate
4.2.1.35
(R)-2-Methylmalate
1.1.1.85
2.3.1.182
Threonine
4.3.1.19
2-Oxobutanoate
Acetyl-CoA
Pyruvate
2.2.1.6
2.2.1.6
(S)-2-Aceto-2-hydroxybutanoate
(S)-2-Acetolactate
1.1.1.86
5.4.99.3
1.1.1.86
5.4.99.3
(R)-3-Hydroxy-3-methyl-2-oxopentanoate
3-Hydroxy-3-methyl-2-oxobutanoate
Pyruvate metabolism
1.1.1.86
1.1.1.86
(R)-2,3-Dihydroxy-3-methylpentanoate
(R)-2,3-Dihydroxy-3-methylbutanoate
4.2.1.9
4.2.1.9
(S)-3-Methyl-2-oxopentanoate
2-Oxoisovalerate
2.3.3.13
(2S)-2-Isopropylmalate
4.2.1.33
2-Isopropylmaleate
4.2.1.33
(2R,3S)-3-Isopropylmalate
2.6.1.42
1.4.1.9
2.6.1.42
1.4.1.9
2.6.1.66
1.1.1.85
L-Isoleucine
L-Valine
(2S)-2-Isopropyl-3-oxosuccinate
spontaneous
4-Methyl-2-oxopentanoate
2.6.1.6
2.6.1.42
1.4.1.9
Valine, leucine and isoleucine degradation
L-Leucine
Data on KEGG graph
Rendered by Pathview

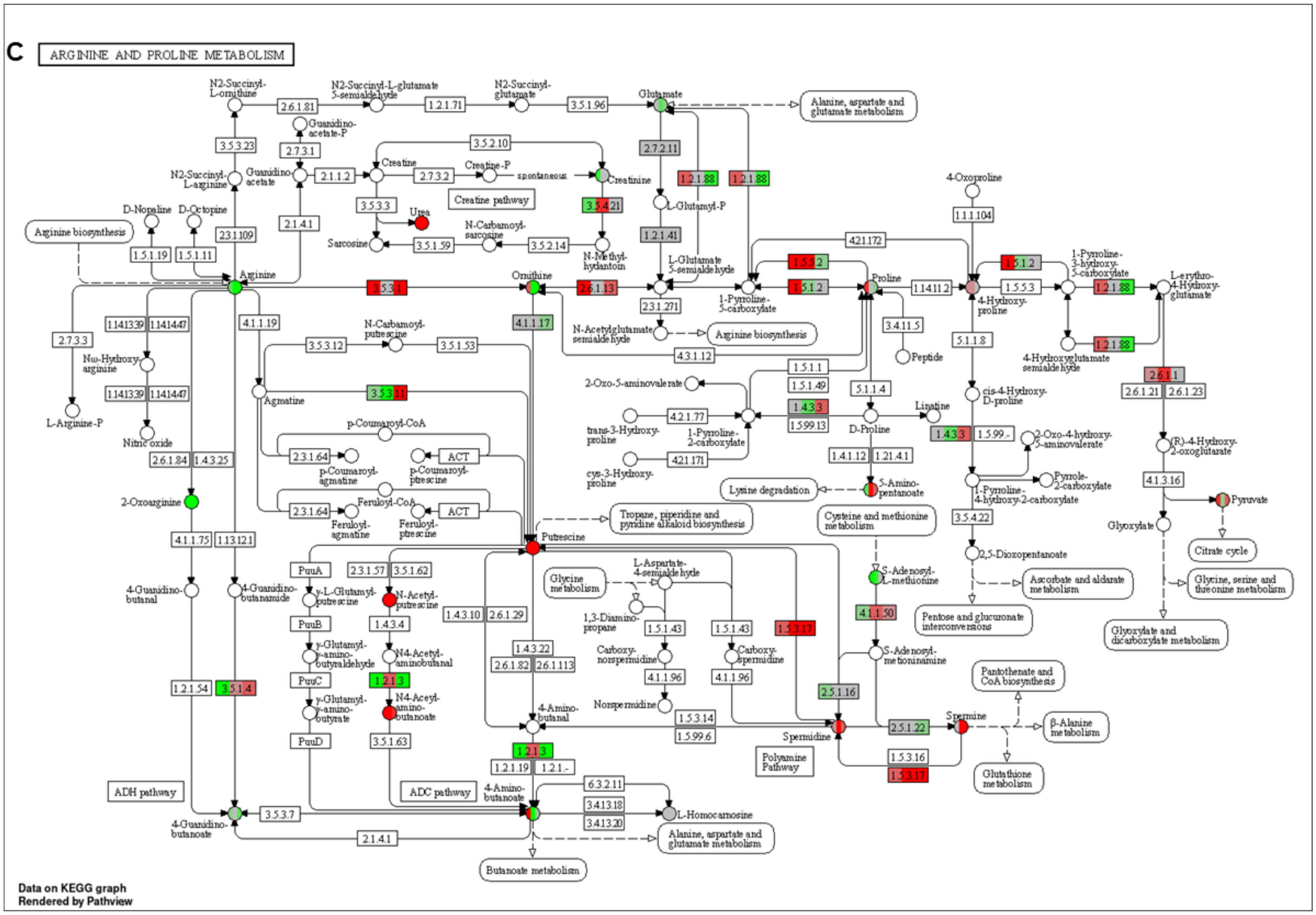

Supplementary Figure 3 Multi-omics analyses of selected amino acid biosynthesis pathways combine transcriptomics and metabolic differences in *stp2*Δ vs. wild type biofilms in the time course of biofilm maturation.

Rectangles represent differential gene expression and circles show differential metabolite abundance in *stp2*Δ vs. wild type cells at 8 h (left), 24 h (middle) and 48 h (right).

(A) Overview of intracellular amino acid abundance show the differential metabolome of *stp2*Δ biofilms (the green arrows indicate the organism-specific pathways within the global map)

(B) Multi-omics analysis of transcriptional and metabolic changes involved in BCAA metabolism

(C) Multi-omics analysis of transcriptional and metabolic changes involved in arginine and proline metabolism

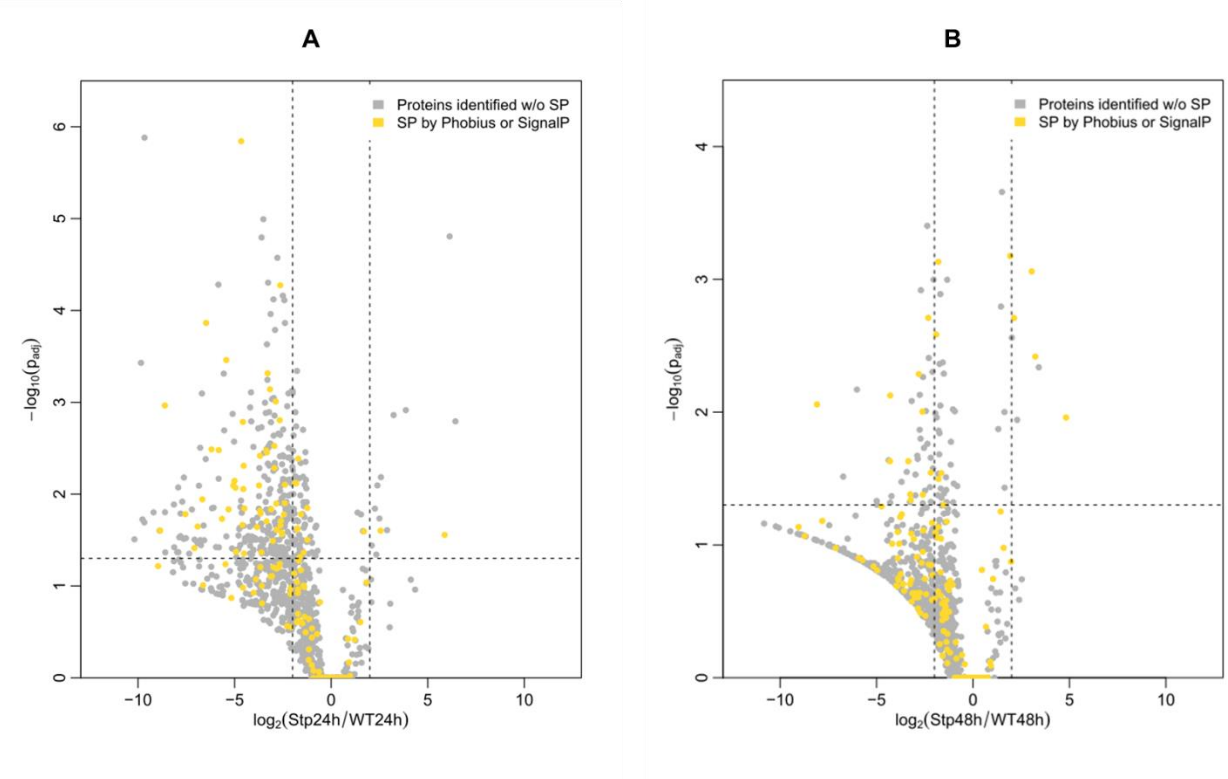

Supplementary Figure 4 Secretome analysis of *stp2*Δ vs WT biofilms

Proteomes of biofilm spent media were compared for stp2Δ against wild type biofilms at (A) 24 h and (B) 48 h incubation. The wild type proteome was higher in cytosolic protein quantity at both time points (volcano plot).

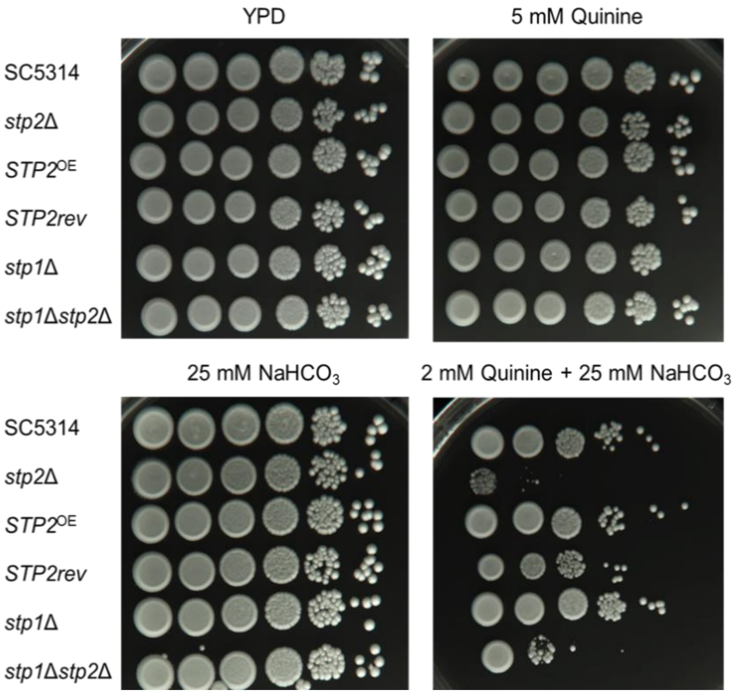

Supplementary Figure 5 Stp2 coordinates the crosstalk between amino acid and glucose starvation

Growth on YPD or the supplementation of 5 mM quinine or 25 mM $NaHCO_3$ was not impaired in any of the tested strains. The combination of quinine and bicarbonate in YPD base medium limit amino acid uptake, which results in lethal amino acid starvation in the *stp2*Δ mutant. Five microliters of serially diluted cells were spotted and cells were grown for 48 h at 30°C.

Supplementary Table 1 Role of Stp2 in *HGT* gene expression during biofilm formation

Member of the *HGT* gene family that were differentially expressed in the *stp2*Δ mutant strain were selected. The cutoff $p_{adj}<0.05$ and $log_2FC \pm 1$ was defined as significant change and was marked in bold.

Gene	Induced at glucose conc. (%) (1)	*stp2*Δ vs. WT 8 h		*stp2*Δ vs. WT 24 h		*stp2*Δ vs. WT 48 h	
		log_2FC	p_{adj}	log_2FC	p_{adj}	log_2FC	p_{adj}
HGT2	2	**1.27**	**0.00**	-0.78	0.41	0.42	0.79
HGT7	2	**3.03**	**0.03**	0.77	0.83	1.31	0.14
HGT8	2	2.01	0.08	-0.52	0.66	-1.03	0.10
HGT10	0.2	2.01	0.27	1.00	0.44	-1.14	0.62
HGT12	0.2	**1.91**	**0.00**	**-1.49**	**0.03**	-0.18	0.88
HGT16	2	0.93	0.00	0.38	0.70	**1.25**	**0.00**
HGT19	2	0.79	0.00	-0.23	0.87	0.38	0.83

Supplementary Table 2 Stp2-mediated amino acid uptake

Essentiality of Stp2 of uptake of amino acids from RPMI medium compared to amino acid sensing that induces Stp2 processing (2).

Amino acids	AA sensing leads to Stp2 processing (from Silao, 2018 (2))	Stp2 is essential for AA uptake
Ala	-	AA secretion
Arg	++	++
Asn	+	++
Asp	++	-
Cys	-	AA secretion
Gln	++	AA secretion
Glu	-	-
Gly	-	No efficient uptake by WT and mutant
His	+	-
Ile	-	++
Leu	-	++
Lys	++	++
Met	-	-
Phe	-	++
Pro	-	Uptake repressed by Stp2 (*stp2*Δ exceeds WT)
Ser	+	++
Thr	+	++
Trp	-	++
Tyr	-	++
Val	-	++

Supplementary Table 3 Core set of differentially abundant proteins in the secretome of *stp2*Δ vs. wild type

Proteins with significantly differential abundance of *stp2*Δ vs. wild type supernatants at both time points.

Related Gene	*stp2*Δ-24h / WT-24h		*stp2*Δ-48h / WT-48h		Description
	log_2FC	*p*adj	log_2FC	*p*adj	
ECM14	-4.59	0.02	-8.09	0.01	Uncharacterized. Has domain(s) with predicted metallocarboxypeptidase activity, zinc ion binding activity and role in proteolysis
ABG1*	-5.07	0.01	-4.31	0.02	Vacuolar membrane protein; depletion causes abnormal vacuolar morphology, cell separation defect, sensitivity to cell wall stress, increased hyphal branching; essential, rat catheter biofilm repressed
LHS1	-2.83	0.02	-4.29	0.01	Protein similar to *S. cerevisiae* Hsp70p; predicted Kex2p substrate; possibly essential, disruptants not obtained by UAU1 method; flow model biofilm repressed
orf19.6553	-6.91	0.02	-3.35	0.02	Membrane-localized protein of unknown function; possibly secreted; fluconazole-induced
GCA2; GCA12	-4.49	0.01	-3.25	0.04	Predicted extracellular glucoamylase; induced by ketoconazole; possibly essential, disruptants not obtained by UAU1 method; promotes biofilm matrix formation; Spider biofilm induced; Bcr1-induced in RPMI a/a biofilms
MNN22; MNN24	-3.69	0.00	-3.23	0.05	Alpha-1,2-mannosyltransferase; required for normal cell wall mannan content
CPY2; PRC3*	-4.53	0.00	-2.81	0.01	Putative carboxypeptidase Y precursor; transcript regulated by Nrg1 and Mig1; regulated by Gcn2 and Gcn4
SSP120	-3.37	0.00	-2.62	0.01	Uncharacterized. Has domain(s) with predicted calcium ion binding activity
ROT2	-8.86	0.02	-2.60	0.04	Alpha-glucosidase II, catalytic subunit, required for N-linked protein glycosylation and normal cell wall synthesis; alkaline downregulated
RNY12*	-4.66	0.00	-2.32	0.00	Has domain(s) with predicted RNA binding, ribonuclease T2 activity
SAP2	5.88	0.03	3.23	0.00	Major secreted aspartyl proteinase; utilization of protein as nitrogen source; role in virulence complicated by URA3 effects; immunoprotective; regulated by growth, albumin, drugs, white cell-type; flow model biofilm induced
PLB1	2.56	0.02	4.82	0.01	Phospholipase B; host cell penetration and virulence in mouse systemic infection; Hog1-induced; signal sequence, N-glycosylation, and Tyr phosphorylation site; induced in fluconazole-resistant strains; rat catheter biofilm repressed

Literature

1. Fan J, Chaturvedi V, Shen SH. Identification and phylogenetic analysis of a glucose transporter gene family from the human pathogenic yeast *Candida albicans*. J Mol Evol. 2002;55(3):336-46.
2. Silao FGS, Ward M, Ryman K, Wallström A, Brindefalk B, Udekwu K, et al. Mitochondrial proline catabolism activates Ras1/cAMP/PKA-induced filamentation in *Candida albicans*. PLOS Genetics. 2019;15(2):e1007976.
